# The new W family reconstructs the evolution of MHC genes

**DOI:** 10.1073/pnas.2122079119

**Published:** 2022-01-24

**Authors:** Jim Kaufman

**Affiliations:** ^a^Institute for Immunology and Infection Research, University of Edinburgh, Edinburgh EH9 3FL, United Kingdom;; ^b^Department of Pathology, University of Cambridge, Cambridge CB2 1QP, United Kingdom

The importance of adaptive immunity has been highlighted by the protection afforded by vaccination in the face of the current coronavirus pandemic. Much has been learned about the origins and subsequent evolution of the antigen-specific receptors used by this crucial arm of the immune response. Among recent discoveries are the early appearance of three lymphocyte lineages in vertebrates with antigen-specific receptors based on leucine-rich repeats in jawless fish but on immunoglobulin (Ig) domains in jawed vertebrates (1), and the proto-RAG transposon which created split genes that could recombine to generate diversity in the three antigen-specific receptors of jawed vertebrates (2). In contrast, there has been little agreement on the origin and subsequent evolution of cell surface molecules encoded by the major histocompatibility complex (MHC), which play central roles in adaptive immunity as the targets of T cell recognition. By discovering the W genes as a proposed intermediate in the evolution of MHC class I and class II genes, the paper by Okamura et al. (3) in PNAS provides a welcome advance.

To appreciate this story, one must understand some aspects of the MHC molecules and the cells that recognize them (4). With well over 10,000 alleles among humans, the classical MHC molecules form the most polymorphic system currently known. Having been discovered as transplantation antigens, their true function is resistance to infectious pathogens and cancers. The high polymorphism is primarily due to a molecular arms race with pathogens, highlighting their importance in resistance to infectious disease. In addition, nonclassical class I molecules have evolved to carry out a wide variety of specialized functions. For example, natural killer (NK) cells of the innate immune system recognize certain classical and nonclassical class I molecules for both immune and nonimmune functions, the latter including placental blood supply for pregnancy (4, 5).

Classical MHC molecules bind peptides within cells for presentation to T cells with T cell receptors (TCRs) composed of α- and β-chains (4, 6). The αβ T cells bearing the coreceptor CD8 recognize classical class I molecules bound to peptides originating primarily in the cytoplasm and nucleus where viruses (and a few intracellular bacteria) replicate. CD8 αβ T cells are cytotoxic T lymphocytes which kill infected cells, preventing the release of new viruses. In contrast, the peptides presented by class II molecules originate largely from intracellular vesicles in contact with the extracellular space where most pathogens can be found, so responses by CD4 αβ T cells are more varied and nuanced, including crucial roles in regulation of most immune responses.

**Fig. 1.**
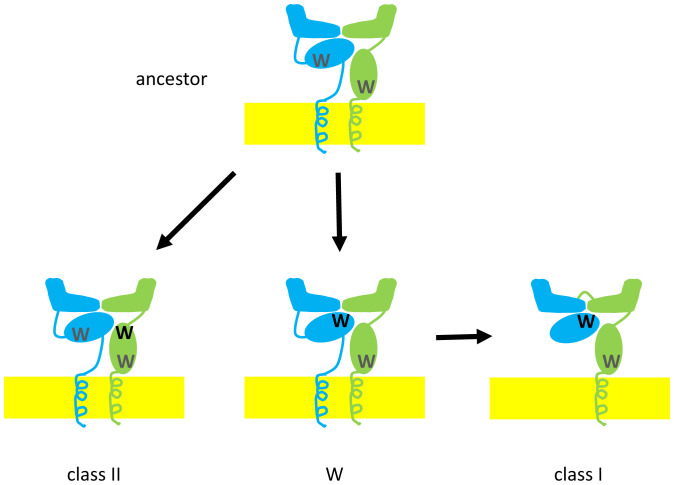
Proposed evolutionary scenario from the ancestral molecule to class II and W molecules, with subsequent evolution from W molecules to class I molecules. The ancestral, class II, and W molecules all have two chains of roughly equal size (α-chain in blue, β-chain in green, membrane in yellow), while the class I molecule has rearranged the domains (β_2_m in blue, heavy chain in blue and then green). The ancestral molecule has nearly invariant tryptophans between the β-sheets of both membrane-proximal Ig-C domains (W in gray), which are maintained in the class II molecule but are replaced by other hydrophobic residues in the W α2 domain and β_2_m. Among the other changes are tryptophans involved in interdomain interaction (W in black): one in the β2 domain of class II molecules and ones in the W α2 domain and β_2_m.

Class I and class II molecules are built of similar protein domains but differ in the organization of these domains (Fig. 1), reflected in the intron–exon structure of their genes (7–11). Class II molecules are heterodimers of α- and β-glycoproteins (encoded by A and B genes), each with a membrane-distal domain and membrane-proximal Ig-constant (Ig-C) domain, a transmembrane (TM) region, and a short cytoplasmic tail. In contrast, class I molecules are composed of one small Ig-C protein, β_2_-microglobulin (β_2_m), in noncovalent association with a large α (or heavy) glycoprotein chain with two membrane-distal domains followed by a membrane-proximal Ig-C domain, a TM region, and a cytoplasmic tail (4, 7–9). For both class I and class II molecules, the two membrane-distal domains together form a pair of broken α-helices atop a platform of β-strands (sometimes called an open-face sandwich or MHC fold) (8, 9). The groove between the α-helices and the β-sheet is where most of the polymorphic positions are found, in which each classical MHC allele binds a different set of peptides (4). The αβ TCRs of T cells and the killer-Ig receptors of human NK cells recognize the peptide and α-helices of the MHC molecules (4, 8, 9).

Although originally envisaged as symmetrical molecules (10), in fact, the Ig-C domain of the class II α-chain and the equivalent β_2_m are wedged under the β-sheet of the membrane-distal domains (8, 9), perhaps to allow the grooves to breathe as they test many peptides for those with appropriate binding. A detailed analysis by coauthor Dijkstra and coworkers (12, 13) identified key attributes of class I and class II molecules, including interdomain contacts, hydrophobic core residues, and sequence indels. Among many examples are three involving tryptophan (single letter code W): one in β_2_m to interact with the membrane-distal α1 and α2 domains of the class I heavy chain, the replacement of one in β_2_m that is otherwise nearly invariant among Ig domains, and one in the membrane-proximal β2 domain of class II molecules to interact with the membrane-distal β1 domain (Fig. 1).

The first sequences of class I and class II molecules revealed their descent from a common ancestor. Once the genes were characterized, a simple evolutionary scenario was evident (10, 11). Since most class II molecules are encoded by A–B gene pairs in opposite transcriptional orientation, an inversion would lead to a class I α-chain gene and a gene encoding an Ig-C domain with a TM region and a cytoplasmic tail, which could give rise to β_2_m by a single mutation. This scenario is supported by the location of the β_2_m gene in the MHC of sharks (14), being cartilaginous fish which are the most primitive living jawed vertebrates, although the β_2_m genes in other jawed vertebrates are located outside of the MHC. An alternative proposal was that class I molecules are ancestral, with the exons encoding peptide-binding domains of a chaperone gene being transferred in front of an exon encoding an Ig-C domain to form the class I α-chain. This tempting scenario was based on proposed sequence similarities between class I molecules and chaperones (15, 16), which became implausible once the completely different structures of the two were determined (11).

The easiest way to determine the evolutionary scenario would be to look at these genes through phylogeny, but there is a gap in the vertebrates between the jawless fish and the jawed vertebrates, where there is a fossil record but no animals surviving to the present day (17). Unfortunately, the important events leading to the emergence of the adaptive immune system of both jawless fish and the jawed vertebrates happened in this gap (11). Without the appropriate living animals, inference based on existing genes and molecules has been the only tool available.

Since discovering the first traces of the W genes some 30 y ago (18), Hashimoto and coworkers (3) have characterized these genes in jawed vertebrates from sharks to salamanders, amassing an enormous amount of data. They show that the WA and WB genes are found in pairs and are expressed as αβ heterodimers, each chain of which has two extracellular domains followed by a TM region and a cytoplasmic tail, just like class II molecules (Fig. 1). In addition, some amino acid positions are in common with class II molecules, including the distinctive pattern of glycines that allows the two TM regions to pack together. However, many key residues are like class I molecules (Fig. 1), including those involved in the interfaces of the extracellular domains such as the tryptophan for which the new group is named, and in the intradomain packing such as the replacement of the nearly invariant tryptophan in common with β_2_m. This loss of tryptophan in β_2_m and the W α2 domain shows that they are both derived from a class II–like ancestor. In addition, phylogenetic analysis groups the W α2 domain with β_2_m, and groups the W β2 domain with the class I α3 domain, showing that the special aspects of class I domain interaction were present in a class II–like heterodimer before the emergence of the class I domain organization.

Every new discovery leads to more questions. What do these current W molecules do? Thus far (3), there is no evidence in existing W molecules for the key residues that coordinate peptide binding in either classical class I or class II MHC molecules, or evidence for high levels of polymorphism. However, there is sequence variation between W genes within species, suggesting various W molecules with different functions. The fact that nonclassical MHC molecules include many class I but few class II molecules may be due to structural reasons (11). The W molecules might provide a test of whether the particular mode of domain interaction contributes to the greater evolvability of class I compared to class II molecules.

Another important question is what the original W molecules did. A radical thought would be to link the function of the original W genes with another enigma, the original function of T cells with TCRs composed of γ- and δ-chains. In mammals, γδ T cells are found largely (but not exclusively) in mucosal tissues, recognizing a wide variety of ligands, including certain nonclassical class I molecules, class II molecules, and butyrophilins (4, 19, 20). Is it possible that γδ T cells acquired an enhanced taste for these various ligands once their original ligands, the W molecules, were accidentally lost in most tetrapod lineages? The tissue distribution of W gene expression might be a first step to examine this conjecture.

And, of course, the origins of the MHC fold and the odd configuration of the two Ig-C domains of MHC molecules remain to be understood. The discovery and characterization of W genes by Okamura et al. (3) provides a stepping stone to answer these questions and many others in the exciting years to come.
